# Abnormalities of intrinsic brain activity in irritable bowel syndrome (IBS): A protocol for systematic review and meta analysis of resting-state functional imaging

**DOI:** 10.1097/MD.0000000000025883

**Published:** 2021-05-28

**Authors:** J. Li, C. Wang, Z.M. Li, B. Fu, Q. Han, M. Ye

**Affiliations:** a Xianning Central Hospital, The First Affiliated Hospital of Hubei University of Science and Technology, Xianning, Hubei; b College of Acupuncture and Orthopedics, Hubei University of Chinese Medicine/Hubei Provincial Collaborative Innovation Center of Preventive Treatment by Acupuncture and Moxibustion, Wuhan, China.

**Keywords:** brain–gut axis, fMRI, irritable bowel syndrome, meta-analysis, ReHo

## Abstract

**Background:**

Irritable bowel syndrome (IBS) is one of the most common functional gastrointestinal (GI) disorders affecting up to 11.5% of the general global population. The brain–gut axis has been shown to play an important role in the pathogenesis of IBS. Several studies confirmed that intrinsic brain abnormalities existed in patients with IBS. But, studies of abnormal regional homogeneity (ReHo) in IBS have reported inconsistent results. The objective of this protocol is to conduct a meta-analysis using the Seed-based *d* mapping software package to identify the most consistent and replicable findings of ReHo in IBS patients.

**Method:**

We will search the following three electronic databases: MEDLINE, EMBASE and Web of Science. The primary outcome will include the peak coordinates and effect sizes of differences in ReHo between patients with IBS and healthy controls from each dataset. The secondary outcomes will be the effects of age, illness severity, illness duration, and scanner field strength. The SDM approach was used to conduct voxel-wise meta-analysis. Whole-brain voxel-based jackknife sensitivity analysis was performed to conduct jackknife sensitivity analysis. A random effects model with Q statistics is used to conduct heterogeneity and publication bias between studies and meta-regression analyses were carried out to examine the effects of age, illness severity, illness duration, and scanner field strength.

**Results:**

The results of this paper will be submitted to a peer-reviewed journal for publication.

**Conclusion:**

This research will determine the consistent pattern of alterations in ReHo in IBS patients.

## 1 Introduction

Irritable bowel syndrome (IBS) is a widespread functional gastrointestinal disorder characterized by recurrent chronic functional gastrointestinal disorder (FGID) with the typical clinical symptoms of recurrent abdominal pain or discomfort, changes in stool characteristics and bowel habits with a global prevalence of about 11.2% of the population.^[1,2]^ It is reported that nearly 50% patients with IBS suffer from psychological–psychiatric and approximately 20–40% of patients accompany with depressive symptoms.^[3,4]^ The interaction of psychosocial factors and gut physiology is the reasons of the pathophysiology of IBS. Studies have shown that anxiety and depression can double the risk of IBS onset.^[5]^ The brain–gut axis is another powerful reason including central nervous system CNS, neuro–endocrine–immune system, hypothalamic–pituitary–adrenal axis, autonomic nervous system, and enteric nervous system and is a bidirectional system enables to communicate between the central nervous system (CNS) and the gut.^[6]^ It plays an important role in IBS by regulating gastrointestinal motility, visceral sensitivity, brain gut peptide secretion, stress response, and cognitive function.^[7]^ But up to now, the neural mechanisms of depressive symptoms in IBS remain unclear. Therefore, further exploration of brain functional changes, such as the intensity of neural activity and functional connectivity (FC) in local brain regions in patients with IBS between specific brain regions with psychological symptoms (such as depression) will help us understand the pathophysiological mechanisms of brain–gut FGID interaction.

Several results from brain imaging studies suggests that IBS is associated with structural and functional changes in the brain.^[8,9]^ Seminowicz found that IBS-D was associated with changes of the posterior parietal cortex (PPC)/middle frontal gyrus (MFG)/bilateral ventral striatum/pregenual anterior cingulate cortex (ACC), pain symptoms with MFG, ventral striatum, ventrolateral prefrontal cortex (vlPFC), Orbitofrontal Cortex (OFC), emotional symptom with MFG, hippocampus and thalamus.^[10]^

Resting-state fMRI (RS-fMRI) is a more important tool to examine brain functional activities of IBS when intestinal tract is at rest state. Based on the amplitude of low-frequency fluctuation (ALFF) and RS-fMRI, Ma^[11]^ applied RS-fMRI and the amplitude of low-frequency fluctuation (ALFF) method in IBS patients and found that the left superior frontal gyrus, right MFG, right hippocampus, right superior temporal pole, and bilateral postcentral gyrus exhibited lower ALFF values, while the left calcarine and left median cingulate exhibited higher ALFF values. There was a significant correlation between duration of disease in IBS and ALFF values in the altered regions. Qi^[12]^ reported that IBS patients had decreased ALFF values in several core default mode network regions and increased ALFF values in the bilateral posterior insula and cuneus.

Regional homogeneity (ReHo) has recently been used as an efficient and reliable neuroimaging marker in the investigation of resting-state regional brain activity in neuropsychiatric disorders such as IBS. ^[13]^ Studies have shown that results of both areas of decreased and increased ReHo are inconsistent.^[13–15]^ In this context, we considered it was appropriate to conduct a meta-analysis to identify the most consistent and replicable ReHo changes in IBS.

## 2 Method and design

This protocol is registered in INPLASY (INPLASY202130108). This is an SRs and MA of clinical studies, so ethical approval is not necessary.

### 2.1 Data sources, study selection, and quality assessment

A comprehensive search of studies published between January 2000 and June 12, 2021 was conducted in the PubMed, Embase, and Web of Science databases using the keywords “Irritable bowel syndrome” OR “Irritable Bowel Syndromes”, OR “Syndrome, Irritable Bowel”, OR “Syndromes, Irritable Bowel”, OR “Colon, Irritable”, OR “Irritable Colon”, OR “Colitis, Mucous”, OR “Mucous Colitides”, OR “Mucous Colitis”, AND “regional homogeneity” OR “ReHo” OR “local connectivity” OR “coherence. Furthermore, the references of the included studies and related review were checked for additional relevant studies. Studies that satisfied the following conditions were included in the meta-analysis:

1. patients had been diagnosed with IBS;
2. ReHo comparison of patients with IBS versus healthy controls was conducted;
3. three-dimensional coordinates (Talairach or Montreal Neurological Institute [MNI]) were reported for the whole-brain ReHo analysis;
4. significant results were reported using thresholds for significance corrected for multiple comparisons or uncorrected with spatial extent thresholds;
5. the study was published in a peer-reviewed English language journal. Datasets were excluded if they explicitly indicated patients with IBS diagnosed with comorbid neurological or psychiatric diseases (i.e., cognitive depression or impairment).

For longitudinal studies, only the baseline data were included. For studies reporting both on- and off-state results, only the off-state datasets were included. In cases where patient datasets overlapped between separate articles, only the dataset with the largest sample size and the most comprehensive information was included. The corresponding author of each included study was contacted via email when additional information was required. The quality of each study selected for this meta-analysis was assessed with a 20-point checklist used in a previous meta-analysis of RS-fMRI studies.^[16]^

The results of electronic searches will be managed using Endnote X9. Two reviewers (Wang C and Li ZM) will review articles identified from different databases according to eligibility criteria independently. Duplicates will be fist removed. A third reviewer (Fu B) made the final decision when there is a disagreement between two researchers. The current study was conducted with reference to the Meta-analysis Of Observational Studies in Epidemiology (MOOSE) guidelines for the meta-analyses of observational studies.^[17]^

### 2.2 Data analysis

#### 2.2.1 Voxel-wise meta-analysis

A meta-analysis of ReHo differences between patients with and healthy controls was conducted using the SDM software package (version 4.31 for Windows) in a standard process (www.sdmproject.com). The SDM approach has been thoroughly described elsewhere.^[18–21]^ We first extracted peak coordinates and effect sizes (e.g., *t*-values) of differences in ReHo between patients with IBS and healthy controls from each dataset. A standard MNI map of the ReHo differences was then separately recreated for each dataset using an anisotropic Gaussian kernel. The mean map was finally generated by voxel-wise calculation of the random-effects mean of the dataset maps, weighted by the sample size, intra-dataset variability, and between-dataset heterogeneity. To optimally balance false positives and negatives, we used the default SDM kernel size and thresholds (full width at half maximum [FWHM] = 20 mm, *P* = .005, uncorrected for FDR, peak height Z = 1, cluster extent = 10 voxels). It should be noted that this FWHM kernel is intended to assign indicators of proximity to reported coordinates rather than to smooth any image that is different in nature.^[18,21]^

If necessary, a subgroup meta-analysis was further conducted.

#### 2.2.2 Jackknife sensitivity analysis

Following preprocessing of the data, a whole-brain voxel-based jackknife sensitivity analysis was performed to test the robustness of the findings by iteratively repeating the same analysis, excluding one dataset each time.^[19,21]^

#### 2.2.3 Analyses of heterogeneity and publication bias

A heterogeneity analysis was conducted using a random effects model with Q statistics to explore unexplained between-study variability in the results. Heterogeneous brain regions were obtained using the default SDM kernel size and thresholds (FWHM = 20 mm, *P* = .005, uncorrected for FDR, peak height Z = 1, cluster extent = 10 voxels).^[18,21]^ In addition, Egger's test was performed using the Stata/SE 12.0 software for Windows (Stata Corp LP, College Station, TX, USA) to assess possible publication bias by extracting the values from statistically significant relevant peaks between patients with IBS and healthy controls.^[22]^

#### 2.2.4 Meta-regression analyses

Meta-regression analyses were carried out to examine the effects of age, illness severity, different disease subtypes (IBS-C OR IBS-D), and scanner field strength, which could potentially influence the analytic results. Statistical significance was determined using a stringent threshold of *P* = .0005 and cluster extent = 10 voxels.^[20,21]^

## 3 Dicussion

Resting-state functional magnetic resonance imaging (rs-fMRI) is a novel approach, which can be conducted to examine brain activity independent of stimulation or goal-directed tasks.^[23–25]^ Regional homogeneity (ReHo), a new indicator of resting-state local brain activity, measures the degree of regional synchronization of fMRI time courses. It calculates the temporal homogeneity of regional brain activity rather than its density, providing information about local coherence or intraregional functional connectivity.^[26]^ Although the exact biological mechanism of ReHo remains unclear, it is thought to reflect the efficiency of coordinated neuronal activity and can be used to assess local alterations in brain function.^[27,28]^

However, findings from these ReHo studies of IBS so far have been inconsistent. In addition, the affected brain regions identified in these studies were diverse with different studies sometimes reporting increased or decreased ReHo in the same brain regions.^[13–15]^ These reported inconsistencies regarding changes in ReHo in patients with IBS can potentially be ascribed to factors such as sample size, illness severity, IBS subtypes, sex and imaging protocols. We hope that the results of this meta-analysis will help to identify the most consistent and replicable ReHo changes in IBS.

## Author contributions

**Conceptualization:** Li ZM.

**Data curation:** Wang C.

**Formal analysis:** Li ZM.

**Funding acquisition:** Ye Mao.

**Methodology:** Ye Mao.

**Project administration:** FU B.

**Resources:** FU B.

**Software:** FU B.

**Supervision:** Han Q, Ye Mao.

**Validation:** Han Q.

**Visualization:** Han Q, Ye Mao.

**Writing – original draft:** Li J.

**Writing – review & editing:** Ye Mao.

## References

[1] Mayer EA. Clinical practice. Irritable bowel syndrome. N Engl J Med 2008;358:1692–9. 18420501 10.1056/NEJMcp0801447 PMC3816529

[2] Lovell RM Ford AC. Global prevalence of and risk factors for irritable bowel syndrome: a meta-analysis. Clin Gastroenterol Hepatol 2012;10:712–21. e714. 22426087 10.1016/j.cgh.2012.02.029

[3] Talley NJ Howell S Poulton R. The irritable bowel syndrome and psychiatric disorders in the community: is there a link? Am J Gastroenterol 2001;96:1072–9. 11316149 10.1111/j.1572-0241.2001.03741.x

[4] Muscatello MR Bruno A Scimeca G Pandolfo G Zoccali RA. Role of negative affects in pathophysiology and clinical expression of irritable bowel syndrome. World J Gastroenterol 2014;20:7570–86. 24976697 10.3748/wjg.v20.i24.7570 PMC4069288

[5] Sibelli A Chalder T Everitt H Workmann P Windgassen S Moss-Morris R. A systematic review with meta-analysis of the role of anxiety and depression in irritable bowel syndrome onset. Psychol Med 2016;46:3065–80. 27605134 10.1017/S0033291716001987

[6] Black CJ Drossman DA Talley NJ Ruddy J Ford AC. Functional gastrointestinal disorders: advances in understanding and management. Lancet (London, England) 2020;396:1664–74. 10.1016/S0140-6736(20)32115-2 33049221

[7] Price DD Zhou Q Moshiree B Robinson ME Verne GN. Peripheral and central contributions to hyperalgesia in irritable bowel syndrome. J Pain 2006;7:529–35. 16885007 10.1016/j.jpain.2005.12.011

[8] Koloski NA Jones M Talley NJ. Evidence that independent gut-to-brain and brain-to-gut pathways operate in the irritable bowel syndrome and functional dyspepsia: a 1-year population-based prospective study. Aliment Pharmacol Ther 2016;44:592–600. 27444264 10.1111/apt.13738

[9] Moloney RD Stilling RM Dinan TG Cryan JF. Early-life stress-induced visceral hypersensitivity and anxiety behavior is reversed by histone deacetylase inhibition. Neurogastroenterol Motil 2015;27:1831–6. 26403543 10.1111/nmo.12675

[10] Seminowicz DA Labus JS Bueller JA. Regional gray matter density changes in brains of patients with irritable bowel syndrome. Gastroenterology 2010;139:48–57. e42. 20347816 10.1053/j.gastro.2010.03.049 PMC2902717

[11] Ma X Li S Tian J. Altered brain spontaneous activity and connectivity network in irritable bowel syndrome patients: a resting-state fMRI study. Clin Neurophysiol 2015;126:1190–7. 25454279 10.1016/j.clinph.2014.10.004

[12] Qi R Liu C Ke J. Intrinsic brain abnormalities in irritable bowel syndrome and effect of anxiety and depression. Brain Imaging Behav 2016;10:1127–34. 26556814 10.1007/s11682-015-9478-1

[13] Ke J Qi R Liu C. Abnormal regional homogeneity in patients with irritable bowel syndrome: a resting-state functional MRI study. Neurogastroenterol Motil 2015;27:1796–803. 26403620 10.1111/nmo.12692

[14] Nan J Zhang L Zheng Q Zhang M Li Z. [Comparison between approximate entropy and regional homogeneity for identification of irritable bowel syndrome based on functional magnetic resonance imaging]. Nan Fang Yi Ke Da Xue Xue Bao 2019;39:1023–9. 31640953 10.12122/j.issn.1673-4254.2019.09.04 PMC6881742

[15] Li J Li GX Guo Y Lu XQ Li L Ding JP. [Regional homogeneity in the patients of irritable bowel syndrome complicated with depression: a resting-state functional magnetic resonance imaging study]. Zhonghua yi xue za zhi 2018;98:196–201. 29374914 10.3760/cma.j.issn.0376-2491.2018.03.008

[16] Iwabuchi SJ Krishnadas R Li C Auer DP Radua J Palaniappan L. Localized connectivity in depression: a meta-analysis of resting state functional imaging studies. Neurosci Biobehav Rev 2015;51:77–86. 25597656 10.1016/j.neubiorev.2015.01.006

[17] Stroup DF Berlin JA Morton SC. Meta-analysis of observational studies in epidemiology: a proposal for reporting. Meta-analysis of Observational Studies in Epidemiology (MOOSE) group. Jama 2000;283:2008–12. 10789670 10.1001/jama.283.15.2008

[18] Lim L Radua J Rubia K. Gray matter abnormalities in childhood maltreatment: a voxel-wise meta-analysis. Am J Psychiatry 2014;171:854–63. 24781447 10.1176/appi.ajp.2014.13101427

[19] Radua J Mataix-Cols D. Voxel-wise meta-analysis of grey matter changes in obsessive-compulsive disorder. Br J Psychiatry 2009;195:393–402. 19880927 10.1192/bjp.bp.108.055046

[20] Radua J Mataix-Cols D Phillips ML. A new meta-analytic method for neuroimaging studies that combines reported peak coordinates and statistical parametric maps. Eur Psychiatry 2012;27:605–11. 21658917 10.1016/j.eurpsy.2011.04.001

[21] Radua J Rubia K Canales-Rodríguez EJ Pomaral-Clotet E Fusar-Poli P Matatix -Cols D. Anisotropic kernels for coordinate-based meta-analyses of neuroimaging studies. Front Psychiatry 2014;5:13. 24575054 10.3389/fpsyt.2014.00013 PMC3919071

[22] Radua J Grau M van den Heuvel OA. Multimodal voxel-based meta-analysis of white matter abnormalities in obsessive-compulsive disorder. Neuropsychopharmacology 2014;39:1547–57. 24407265 10.1038/npp.2014.5 PMC4023155

[23] Tillisch K Labus JS. Advances in imaging the brain-gut axis: functional gastrointestinal disorders. Gastroenterology 2011;140:407–11. e401. 21167161 10.1053/j.gastro.2010.12.014 PMC4098933

[24] Biswal BB Mennes M Zuo XN. Toward discovery science of human brain function. Proc Natl Acad Sci U S A 2010;107:4734–9. 20176931 10.1073/pnas.0911855107 PMC2842060

[25] Lee MH Smyser CD Shimony JS. Resting-state fMRI: a review of methods and clinical applications. AJNR Am J Neuroradiol 2013;34:1866–72. 22936095 10.3174/ajnr.A3263 PMC4035703

[26] Zhong Y Zhang R Li K. Altered cortical and subcortical local coherence in PTSD: evidence from resting-state fMRI. Acta Radiol 2015;56:746–53. 24973255 10.1177/0284185114537927

[27] Späti J Hänggi J Doerig N. Prefrontal thinning affects functional connectivity and regional homogeneity of the anterior cingulate cortex in depression. Neuropsychopharmacology 2015;40:1640–8. 25598428 10.1038/npp.2015.8 PMC4915268

[28] Philip NS Kuras YI Valentine TR. Regional homogeneity and resting state functional connectivity: associations with exposure to early life stress. Psychiatry Res 2013;214:247–53. 24090510 10.1016/j.pscychresns.2013.07.013 PMC3849340

